# Self-Polarized P(VDF-TrFE)/Carbon Black Composite Piezoelectric Thin Film

**DOI:** 10.3390/polym15204131

**Published:** 2023-10-18

**Authors:** Lavanya Muthusamy, Balaadithya Uppalapati, Samee Azad, Manav Bava, Goutam Koley

**Affiliations:** 1 Holcombe Department of Electrical and Computer Engineering, Clemson University, Clemson, SC 29634, USA; buppala@g.clemson.edu (B.U.); sameea@g.clemson.edu (S.A.); gkoley@clemson.edu (G.K.); 2 Department of Physics and Astronomy, Clemson University, Clemson, SC 29634, USA; mbava@g.clemson.edu

**Keywords:** P(VDF-TrFE) thin films, flexible substrate, piezoelectric polymer, carbon nanocomposites, d_33_ coefficient, self-poling

## Abstract

Self-polarized energy harvesting materials have seen increasing research interest in recent years owing to their simple fabrication method and versatile application potential. In this study, we systematically investigated self-polarized P(VDF-TrFE)/carbon black (CB) composite thin films synthesized on flexible substrates, with the CB content varying from 0 to 0.6 wt.% in P(VDF-TrFE). The presence of –OH functional groups on carbon black significantly enhances its crystallinity, dipolar orientation, and piezoelectric performance. Multiple characterization techniques were used to investigate the crystalline quality, chemical structure, and morphology of the composite P(VDF-TrFE)/CB films, which indicated no significant changes in these parameters. However, some increase in surface roughness was observed when the CB content increased. With the application of an external force, the piezoelectrically generated voltage was found to systematically increase with higher CB content, reaching a maximum value at 0.6 wt.%, after which the sample exhibited low resistance. The piezoelectric voltage produced by the unpoled 0.6 wt.% CB composite film significantly exceeded the unpoled pure P(VDF-TrFE) film when subjected to the same applied strain. Furthermore, it exhibited exceptional stability in the piezoelectric voltage over time, exceeding the output voltage of the poled pure P(VDF-TrFE) film. Notably, P(VDF_TrFE)/CB composite-based devices can be used in energy harvesting and piezoelectric strain sensing to monitor human motions, which has the potential to positively impact the field of smart wearable devices.

## 1. Introduction

Recent advancements in flexible piezoelectric materials have paved the way for the potential realization of self-powered flexible devices in wearable electronics and other fields [1,2,3]. These materials have the ability to efficiently convert various forms of mechanical force into electrical power, eliminating the need for external power sources. Among the materials explored for this purpose, polyvinylidene fluoride (PVDF) and its copolymer P(VDF-TrFE) have stood out for their outstanding properties, including high piezoelectric coefficients, improved crystallinity, enhanced remnant polarization, and superior temperature stability [4,5,6,7]. P(VDF-TrFE) is classified as a ferroelectric polymer with an inherent β phase structure, which is achieved by changing the TrFE molar ratio with respect to PVDF [8,9]. However, P(VDF-TrFE) has a relatively low piezoelectric coefficient [9,10,11]. To enhance the piezoelectric coefficient and optimize energy conversion efficiency, aligning the dipoles within P(VDF-TrFE) films in a specific orientation is crucial. Traditional methods, such as polarization under a high electric field [3,12], electrospinning [13,14], corona poling [15,16], or thermal poling [17], have been employed to achieve this self-alignment. However, these methods often involve complex processing steps, are time-consuming, and may lead to dielectric breakdown within the film when a high electric field is applied, leading to reduced yield and constraints on practical implementation.

Recently, researchers have explored the fabrication of self-poled polymer/composite films using various techniques, such as Langmuir–Blodgett deposition [18], casting [19,20,21], and the incorporation of nanofillers such as ZnO, Yb3+, BTO, CNT, MXene, and graphene oxide [22,23,24,25,26,27,28,29]. In these approaches, nanofillers play a crucial role as nucleating agents, inducing the alignment of polymer molecular chains toward the nanofiller surface. This alignment is facilitated by the strong interaction between the negatively charged fluorine atoms and the positively charged hydrogen atoms originating from the hydrophilic tail group (hydroxyl group) on the nanofiller surface. As a result, the seed layer aligned perpendicular to the substrate, and subsequent layers were arranged in an upward fashion. This alignment not only simplifies the fabrication process but also enhances versatility, scalability, and cost-effectiveness while reducing energy consumption.

Among carbon-based nanofillers, carbon black is considered a promising candidate owing to its excellent electrical properties, high chemical and thermal stability, abundant availability, affordability, and a straightforward synthesis process [30]. The presence of –OH groups on the surface of carbon black facilitates the uniform arrangement of P(VDF_TrFE) molecular backbones. Previous studies have demonstrated significant enhancements in open-circuit voltage and harvested power density when CB is incorporated into polymer films. Wu et al. [31] introduced CB into poly(vinylidene fluoride-co-hexafluoropropylene) (PVDF-HFP) and observed a remarkable enhancement in the open-circuit voltage and harvested power density of the composite films after poling, with improvements of 104% and 364%, respectively, compared to pristine PVDF-HFP. Similarly, Alamusi et al. [32] fabricated P(VDF-TrFE)/CB composite films, achieving an open-circuit voltage of 10.07 V for the 0.8 wt.% poled CB composite film, which exceeded the 5.63 V obtained via the pure poled P(VDF-TrFE) film by a factor of 1.7. However, these studies mainly focused on relatively thick films prepared through post-poling treatments, which can be labor-intensive. Furthermore, comprehensive characterization and long-term performance evaluation were often lacking, which are critical aspects for practical applications.

In this study, we systematically incorporated carbon black into P(VDF-TrFE) at varying compositions (0–1 wt.%) to prepare self-poled P(VDF-TrFE)/CB composite films using a simple spin-coating process. Our investigation incorporated an in-depth analysis of the influence of CB composition on the P(VDF-TrFE) matrix and the formation of the β phase, contributing to high piezoelectric output. To accomplish this, we utilized a range of characterization techniques, including optical microscopy, atomic force microscopy (AFM), X-ray diffraction (XRD), and Fourier-transform infrared (FTIR) spectroscopy. Furthermore, we measured the piezoelectric performance and piezoelectric coefficient (d_33_) of the resulting CB composite films under poled and unpoled conditions. The unpoled 0.6 wt.% CB composite film exhibited exceptional performance, superior to that of the unpoled composite films, and maintained its piezoelectric performance consistently over time. These findings significantly exceeded the performance of the poled films and were confirmed in a 7-day study.

## 2. Experimental Details

### Synthesis of P(VDF_TrFE)/CB Composite Films

P(VDF-TrFE) (55:45 molar ratio) powder was purchased from Piezotech (Arkema Group, Wetherby, UK). The CB powder, with a particle size of 30 nm and a specific surface area of 254 m^2^/g, was obtained from the Cabot Corporation (Boston, MA, USA). In our experiments, six different weight percentages (ranging from 0 to 1 wt.%) of CB powder and P(VDF-TrFE) were dissolved in N, N-dimethylformamide (DMF) for 12 h at 40 °C to obtain a homogeneous solution.

**Figure 1:**
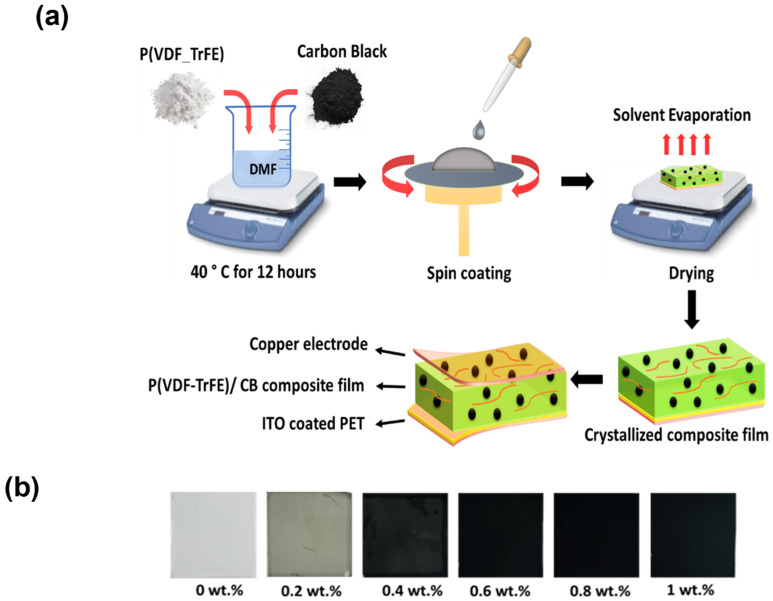
(**a**) Schematic representation for the fabrication process of the flexible P(VDF-TrFE)/CB composite films. (**b**) Photo images of the fabricated P(VDF-TrFE)/CB composite films on glass substrates with CB content varying from 0 wt.% to 1 wt.%.

Figure 1a depicts a schematic of the fabrication process for P(VDF-TrFE)/CB composite films. We used ITO-coated PET substrates in which the ITO layer served as the bottom electrode. Before spin coating, the PET substrate was cut into 2 × 2 cm pieces and cleaned with acetone and IPA. Then, the solution was spin-coated onto the ITO-coated PET substrate at 3000 RPM for 30 s to achieve a ~5 µm thickness film. Subsequently, the film was placed on a hotplate and baked at 60 °C for 1 h to remove the solvent. Following this, the film was annealed at 140 °C for 2 h at room temperature to enhance its crystallinity, as previous studies have reported that crystallization occurring at 140 °C yields the most stable form of the β-phase with a high dielectric constant [1,5,7].

After the sample was cooled to room temperature, an adhesive copper tape was placed on the composite film to establish the top electrode. As seen in Figure 1b, the films gradually became darker with an increasing weight percentage of CB in the polymer matrix. The thickness of the prepared films was measured via a profilometer (Tencor AS-200) and found to be 5, 5.1, 5.18, 5.3, 5.4, and 5.5 µm of 0, 0.2, 0.4, 0.6, 0.8, and 1wt.% of CB, respectively. For poling the samples, a 100 V/µm electric field was used for an hour using a high-power DC supply (Hewlett Packard 6515A, USA). The poling voltage was increased in steps of 100 V/µm every 10 min to avoid electrical breakdown due to sudden and non-uniform charge accumulation [7].

## 3. Results and Discussion

### Material Characterization

Following synthesis, the surface morphology of the resulting P(VDF-TrFE)/CB composite films was examined. Optical microscope images were captured using Olympus BX41M-LED at 50× magnification. In addition, atomic force microscopy (AFM, Veeco Dimension 3100) operated in tapping mode was employed to provide higher-resolution images of the composite films, and the AFM images were subsequently processed using the dedicated AFM software. To determine the effect of blending CB to the P(VDF-TrFE) polymer matrix and the crystallinity of the P(VDF-TrFE)/CB composite films, X-ray diffraction (XRD) measurements (Rigaku Smart Lab system) were made on the composite films (with CB varying from 0 to 1 wt.%) using Cu Kα radiation (wavelength 15.406 nm) in the 2θ range from 5° to 90° with a step size of 0.5°.

Furthermore, FTIR spectra were measured (model no: Thermo Scientific Nicolet380) in the range of 4000–400 cm^−1^ with 64 scans at a 4 cm^−1^ resolution. The percolation threshold (transition point from insulator to conductor) and conductivity measurements were conducted using a Source Measure Unit (SMU, B2902A, Keysight, Santa Rosa, CA, USA). To measure the piezoelectric voltage output of the composites, they were excited by an external shaker (LDS V201, Brüel & Kjær, London, UK), and the voltage waveforms were recorded using a digital storage oscilloscope (DSO 5102P, Hantek, Qingdao, China).

**Figure 2:**
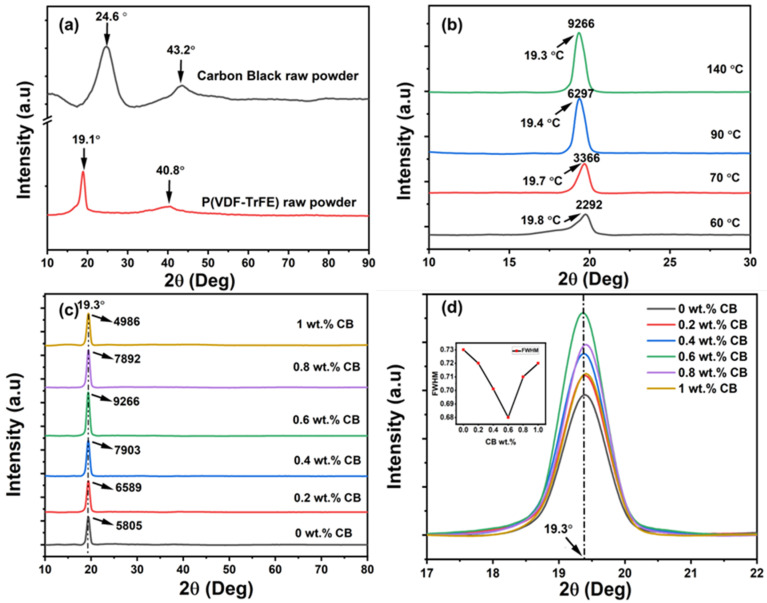
XRD spectra for (**a**) raw carbon black and P(VDF-TrFE) powder. (**b**) Temperature vs. 2θ variation for 0.6 wt.% CB composite film, varying from 60 to 140 °C. (**c**) P(VDF-TrFE)/CB composite films, varying from 0 to 1 wt.%, over the scan range of 10° to 90° to study the diffraction peaks from (110/200) planes of the P(VDF-TrFE) β-phase formation at 19.3°. (**d**) A magnified view of the XRD spectra in the range of 17–22° for demonstrating the effect of adding CB. The inset shows FWHM for different CB composite films, varying from 0 to 1 wt.%.

Figure 2a shows the XRD spectrum of raw carbon black and P(VDF-TrFE) powder with a molar ratio of 55:45. Raw P(VDF-TrFE) powder exhibits a prominent β-phase peak (110/200) at 2θ = 19.1°, which experimentally confirms the presence of the TrFE unit in more than 20%, directly crystallized into the β-phase within the polymer [33,34]. Additionally, a broader peak at 40.8° corresponds to the diffraction plane (111/201), further validating the presence of the β-phase in the polymer matrix. Notably, no significant peak was observed at 2θ = 18.27°, corresponding to the (100) crystal planes of the α-phase of the P(VDF-TrFE) polymer [35]. In the XRD results for raw CB powder, the (002) diffraction peak appears at 24.5°, along with a broader and weaker (001) peak at 43°, consistent with previous reports [36,37].

Figure 2b shows the XRD diffraction peaks of the 55/45 copolymer films crystallized at different temperatures (T_cr_) to determine the optimum crystallization temperature for these films. Typically, crystallization begins when the film is subjected to a temperature above the Curie temperature (T_c_ = 60 °C for 55:45 mol ratio). We found that at a crystallization temperature (T_cr_) of 60 °C for P(VDF-TrFE), the maximum diffraction angle appeared at 2θ = 19.8°, which is close to 20.12°, corresponding to the Bragg diffraction of (110)/(200) of the β-phase. However, the diffraction peak intensity of the composites annealed at 60 °C is relatively small compared to peak intensities at other crystallization temperatures and existed within the amorphous region. As the crystallization temperature (T_cr_) increased, the amorphous region gradually disappeared, and the peak intensities became narrower and sharper. Meanwhile, the diffraction angle (2θ) shifted to a lower value, changing from 20.12° to 19.3° (with an increase in d-spacing from 4.46 to 4.59 Å) as displayed in Figure 2b. This shift was due to the ferro-to-paraelectric transition occurring during the crystallization phase, which changed the trans-planar conformation (TT) to the trans-gauge conformation (TG). Consequently, it shifted the prominent β-phase peak from 2θ = 20.12° to lower values and increased peak intensities as the crystallization temperature (T_cr_) increased [38]. Moreover, the full width at half-maximum (FWHM) value, which is directly related to the degree of crystallinity, gradually improved as the annealing temperature increased from 60 to 140 °C. This observation clearly indicates an enhancement in the electroactive polar β-phase content in the composite films.

We investigated the pure P(VDF-TrFE) film under various annealing temperatures (e.g., 60 °C, 80 °C, and 100 °C) and found a similar trend (see Supplementary Figure S1). From the XRD results, we can conclude that annealing the composite at 140 °C results in optimal crystalline quality, corresponding to the most stable β-phase in the film. Table 1 summarizes the diffraction peak angles (2θ), interplanar spacing (D), FWHM values for the prominent β-phase peak, and corresponding peak intensities of the 0.6 wt.% CB film annealed at various crystallization temperatures. Figure 2c displays the XRD pattern of P(VDF-TrFE)/CB composite films, ranging from 0 to 1 wt.%, within the scan range of 10° to 90°. An analysis of the XRD pattern reveals that a well-defined diffraction peak appears at an angle of 19.3°, corresponding to the (110)/(200) planes of the β-phase, which comprises all-trans TT conformation [39].

In particular, we did not observe any significant peak for CB due to its amorphous nature and the low percentage of CB present in the polymer matrix. This clearly indicates that the addition of CB does not significantly alter the crystalline structure of P(VDF-TrFE). Figure 2d shows the magnified view of XDR spectra of different composite films in the scan range of 17°–22°, varying from 0 to 1 wt.%. With an increase in CB content from 0 to 0.2, 0.4, 0.6, 0.8 wt.%, and 1 wt.% in the composite, XRD peak intensity varied from 5805 to 6589, 7903, 9266, 7892, and finally to 4986, respectively. Above 0.6 wt.%, a reduction in the diffraction peak intensity and an increase in FWHM were observed, as seen in the inset image of Figure 2d, indicating that the best crystalline properties were achieved for the 0.6 wt.% composition. However, when the amount of CB percentage exceeds the optimum amount of 0.6 wt.%, the aggregation created by CB reduces the formation of the β-phase in the polymer. The homogeneous dispersion of carbon black particles within the polymer matrix is a major factor in the increased intensity. Similarly, Yaseen et al. [40] observed the same trend with P(VDF-TrFE)/reduced graphene oxide (rGO) composite films. The observed intensities for all composite films, along with interplanar spacing (D) and FWHM, are tabulated in Table 2.

**Figure 3:**
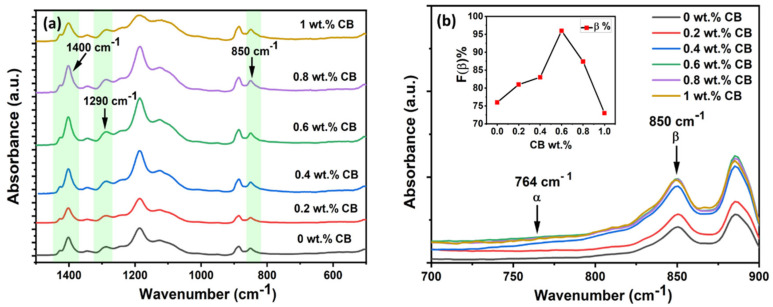
FTIR spectra of (**a**) P(VDF−TrFE)/CB composite films varying from 0 to 1 wt.%. The dashed lines indicate β-phase characteristic peaks at the wavenumber 850 cm^−1^, 1290 cm^−1^, and 1400 cm^−1^. (**b**) A magnified view of the FTIR spectra between 700 and 900 cm^-1^ representing the intensity change in absorbance peaks of the α and β phase upon the addition of CB into P(VDF−TrFE). The inset shows the percentage of β-phase crystallinity vs. different CB wt.%.

FTIR characterization of the films was also conducted to confirm the XRD results and study the interaction between the nanoparticle’s surface and P(VDF-TrFE). The formation of the beta phase in P(VDF-TrFE) was identified by examining three important absorbance peaks (850 cm^−1^, 1288 cm^−1^, and 1400 cm^−1^) in the FTIR spectra. The 1400 cm^−1^ band corresponds to the -CH_2_ wagging vibration, while the 1288 cm^−1^ and 850 cm^−1^ bands are attributed to the -CF_2_ symmetric stretching, with dipoles parallel to the b-axis [41]. Figure 3a illustrates the FTIR spectra of P(VDF-TrFE)/CB thin films ranging from 0 to 1 wt.%, and the prominent peaks for the β-phase are consistent for all CB compositions within the IR detection limit (1400 cm^−1^ to 400 cm^−1^). This indicates that the crystalline quality remains unaffected by the incorporation of CB nanoparticles.

**Figure 4:**
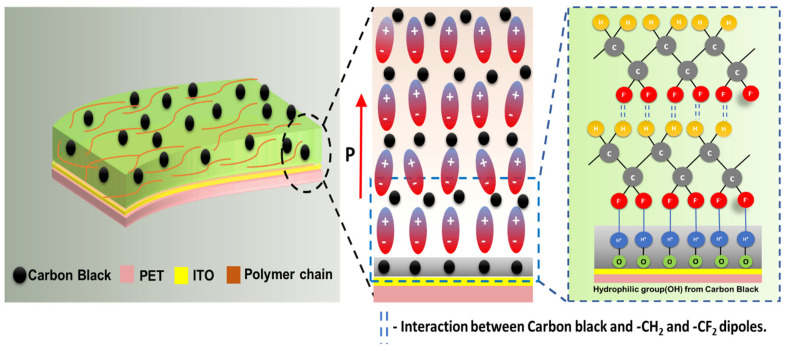
Schematic representation of electrostatic interaction between PVDF−TrFE chains and CB indicating the formation of self-aligned dipoles. The enlarged view shows possible intermolecular hydrogen bonding between positively charged hydrogen atoms from the hydrophilic group(–OH) of carbon atoms and negatively charged fluorine atoms.

The P(VDF-TrFE) film and other CB composite films show increasing intensity in all observed peaks (1400 cm^−1^, 1290 cm^−1,^ and 850 cm^−1^) as the CB composition is increased up to 0.6 wt.% beyond which it remains constant or even decreases somewhat. This increase is attributed to the specific interaction between hydroxyl (–OH) groups found on the surface of carbon nanofillers and CF_2_ segments of P(VDF-TrFE). This interaction becomes more pronounced with an increase in CB content, reaching a maximum of 0.6 wt.%. Figure 3b provides a magnified view of the peaks in the scan range of 750 cm^−1^ to 900 cm^−1^, allowing for the calculation of the percentage of β-phase crystallization F(β) by measuring the absorbance intensity of the β-phase and α-phase using the following formula: [42]

(1) $$F(\beta )=\frac{X_{\beta }}{X_{\alpha }+X_{\beta }}=\frac{A_{\beta }}{(K_{\beta }/K_{\alpha })\;A_{\alpha }+A_{\beta }\;}\;=\frac{A_{\beta }}{1.26\;A_{\alpha }+A_{\beta }}$$

where *X_α_* and *X_β_* are the crystalline mass fractions of the α and β-phases, and *A_α_* and *A_β_* correspond to absorbance at 764 cm^−1^ and 850 cm^−1^, respectively [40,43]. The values of the absorption coefficients result in *K_β_*/*K_α_* = 1.26. FTIR measurements were carried out to calculate F(β) for various CB composites, and the results are plotted in the inset of Figure 3b and listed in Table 2. As shown in Figure 3b inset, F(β) is approximately 76% for the pure P(VDF-TrFE) film prepared and crystallized at 140 °C. It increases monotonically until it reaches a maximum of ~97% for the 0.6 wt.% CB composition. However, at higher CB contents, the incorporation of carbon particles has a negative impact on the β-phase formation, leading to a reduction in the percentage of beta crystallinity to 75% [44]. These results are in good agreement with the XRD results discussed earlier, indicating that the best film quality is obtained for 0.6 wt.% CB. Moreover, the broadened absorbance peak occurring between 3600 and 3400 cm^−1^ implies the formation of intermolecular hydrogen bonding between -CF2- dipoles and the hydrophilic groups from CB, as well as the remaining oxygen-containing groups from CB [40,44]. The OH stretching is much stronger for the 0.6 wt.% concentration than at the lower concentration (0 wt.%), clearly indicating that more –OH groups of CB form bonds with the most negatively charged fluorine atoms in the P(VDF-TrFE) molecular chains (see Supplementary Figure S2). This explains the possible electrostatic interaction between positively charged hydrogen atoms drawn from the hydrophilic tail group (–OH) of carbon black and negatively charged fluorine atoms from P(VDF-TrFE), as seen in Figure 4.

Such bonding is favored due to the large electronegativity differences between the atoms involved.

The hydrophilic nature of the –OH groups causes the seed layer to align perpendicularly to the substrate. As shown in the enlarged view in Figure 4, other induced dipoles resulting from hydrogen intermolecular bonding can interact with each other within subsequent polymer matrices. This interaction occurs between carbon black and –CH_2_ dipoles, leading to local alignment during crystallization [24,25,28,45]. The interaction between –CF_2_–CH_2_– dipoles and carbon black can be confirmed by investigating the –CH_2_ symmetric and asymmetric stretching vibrational bands at 3012 cm^−1^ and 2978 cm^−1^ in the FTIR spectra, which are not associated with any other vibrational bands. These vibrational bands shifted to lower frequencies as the CB loading increased; meanwhile, the absorbance peak intensity increased with respect to carbon weight percentage [20,40] (see details in Supplementary Figure S2).

**Figure 5:**
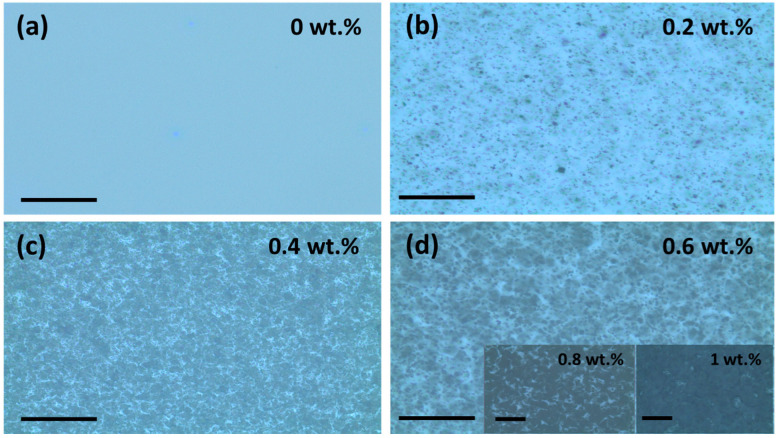
Optical images of P(VDF-TrFE)/CB composite films (50× magnification) for (**a**) 0 wt. %, (**b**) The 0.2 wt.%, (**c**) 0.4 wt.%, and (**d**) 0.6 wt.% CB content. The inset shows the optical images of 0.8 and 1 wt.% CB composite films. All composite films used in this characterization were annealed at 140 °C. The scale bar is 500 µm for all composite films.

Figure 5 shows optical images of different CB composite films at 50× magnification with a 500 µm scale bar, ranging from 0 to 1 wt.%. In Figure 5a, the smooth surface of the pure P(VDF-TrFE) film is illustrated, while Figure 5b–d show the distribution of CB particles within the polymer matrix. These CB particles are clearly noticeable in optical images, consistent with an earlier report [46]. As shown in Figure 5b, CB particles diffuse randomly and form tiny agglomerates at lower CB wt.%. As the CB fraction increases, these agglomerates become larger and larger, ultimately creating a conductive path between them. However, at very high CB content (~1%), structured agglomerates are no longer formed. Instead, the polymer matrix becomes saturated, resulting in a continuum of particles, indicated by a uniform dark color, as seen in the inset of Figure 5d. We also studied the surface morphologies of the composite films using AFM to analyze nanoscale variations in surface roughness caused by CB incorporation.

**Figure 6:**
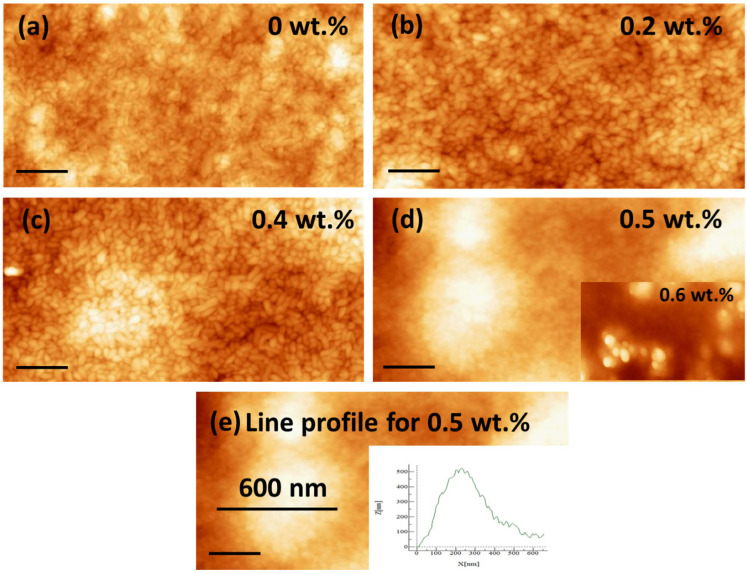
Surface morphology AFM images (5 × 2.5 µm) of different CB films annealed at 140 °C for (**a**) 0 wt.% (**b**) 0.2 wt.% (**c**) 0.4 wt.% (**d**) 0.5 wt.%, and (**e**) Line profile for 0.5 wt.%. The scale bar is 200 nm for all composite films. The RMS roughness is determined as 5.2, 8, 21, and 37 nm, respectively.

Figure 6 displays AFM images (5 × 2.5 µm) of different films with CB concentrations ranging from 0 to 0.6 wt.%. These images generally reveal uniformly distributed “rice grain”-like crystallites with dimensions in the tens of nanometers. Previous studies have also reported similar rice grain domains for pure P(VDF-TrFE) films annealed at 140 °C [47]. In contrast, when the films were subjected to temperatures near their melting point (T_m_ = 153 °C), we observed more interconnected nanofiber-like crystallites, characterized by a higher roughness of approximately 36.8 nm for the 0 wt.% CB sample (see Supplementary Figure S3). In general, nanofillers tend to increase the surface roughness [48], as observed in these P(VDF-TrFE)/CB composites annealed at 140 °C. The lowest roughness, approximately 5 nm, was observed for the 0 wt.% CB film. This roughness increased progressively with increasing CB content: 5.2 nm, 8 nm, 21 nm, and finally 37 nm for the CB compositions of 0.2 wt.%, 0.4 wt.%, 0.5 wt.%, and 0.6 wt.%, respectively. A 600 nm line profile across an elevated carbon aggregate “island” region on the 0.5 wt.% CB film is shown in Figure 6e, which indicates a height and span of ~400 nm for the island.

## 4. Electrical Characterization

### 4.1. Resistivity Measurement

**Figure 7:**
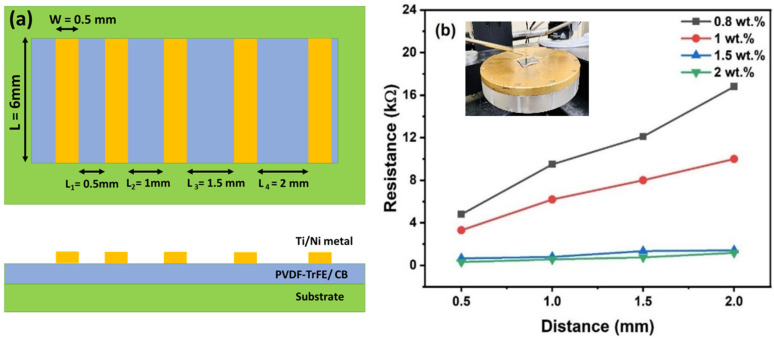
(**a**) Schematic diagram showing parallel metal contacts with varying gaps for TLM measurements to determine the sheet resistance of the films with higher CB content. (**b**) Measured resistance vs. gap for different composite films with CB content varying from 0.8 to 2 wt.%.

Carbon black is conductive in nature, and its introduction in P(VDF-TrFE)/CB composites can help tune the resistivity of the insulating polymer (apart from boosting its energy harvesting performance), which has been gaining more attention in recent years. To find the optimal amount of CB for the composites (which provides maximum output voltage without making the film too conducting), the electrical characteristics of these films, with varying CB content, were systematically characterized. As shown in Figure 7a, four contacts of 0.5 mm × 6 mm were established with varying gaps of (0.5 mm, 1 mm, 1.5 mm, and 2 mm) to perform transmission line-type measurements. The resistances for various CB % for the four gaps are shown in Figure 7b, with the inset showing a close-up of the measurement setup with the sample. The contact resistance (R_c_) and sheet resistance (R_s_) of the composites were estimated from the least square fits using MATLAB programming (details provided in Supplementary Information, Figure S4).

We found that composite films with CB ranging from 0 to 0.6 wt.% are insulating, which indicates that the CB agglomerates are spatially well separated within the polymer matrix, and a percolation path does not exist [30]. Beyond 0.6 wt.% of CB, the P(VDF-TrFE)/CB composites became slightly conductive because of the barrier tunneling effect between the polymer chains and CB agglomerates, and a finite resistance was measured. The sheet resistance (R_s_) was determined to be 46 kΩ/□ for 0.8 wt.% CB content, which was reduced to 12 kΩ/□ for 1 wt.% CB. Further, R_s_ decreases sharply with CB % until 1.5 wt.%, beyond which it reaches a saturation value of ~3.3 kΩ/□. Due to significant film conductivity, the piezoelectrically generated voltage could only be measured for CB content up to 0.6 wt.%, which is discussed below.

### 4.2. Experimental Setup

**Figure 8:**
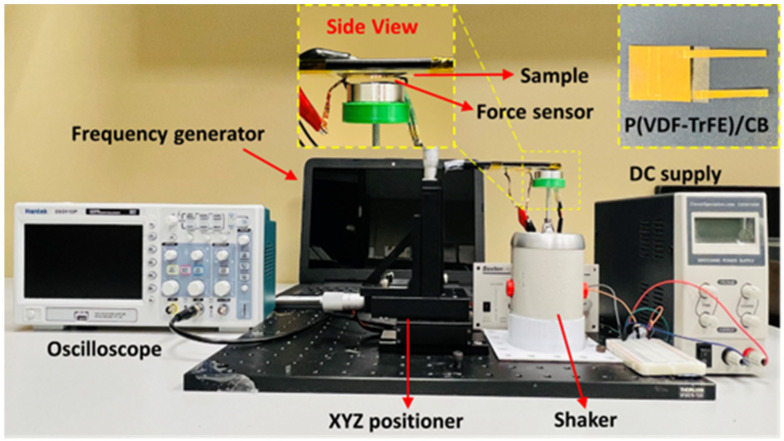
Schematic representation of the experimental setup for piezoelectric output voltage measurement using periodic mechanical force generated by a shaker. The top-left inset shows a magnified view of the sample and force-sensing resistor, while the top−right inset shows the optical image of a fabricated energy harvester device with electrical contacts to the top and bottom surfaces of the sample.

The experimental setup in Figure 8 was utilized to measure the piezoelectrically generated output voltage from the fabricated P(VDF-TrFE)/CB composite films for various CB content. In these experiments, an adjustable extended fixed arm attached to an XYZ positioner was used to mount the device at the bottom surface, which was pressed periodically using a cylinder attached to a shaker, as shown in Figure 8, and its insets show the image of fabricated PENG and Force sensing resistor (FSR) attachment to the shaker. The output voltage transients were measured and recorded using a digital storage oscilloscope as the fabricated composite devices were subjected to stress generated by the mechanical shaker (LDS, V201). A force-sensing resistor (FSR) was carefully calibrated using a reference chart provided by the manufacturer (details provided in Supplementary Information Figure S5) and placed on top of the cylinder to measure the applied periodic force on the film.

### 4.3. Piezoelectric Measurement

**Figure 9:**
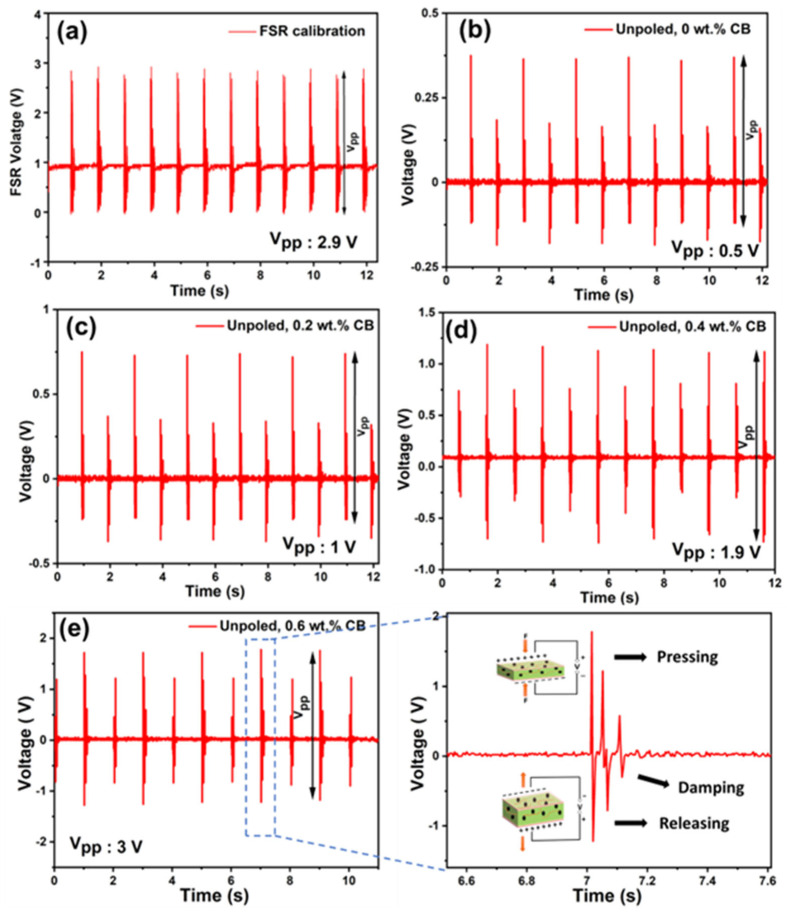
Frequency−dependent calibrated output voltage of different unpoled P(VDF−TrFE)/CB composite films under 6 N applied force with 1 Hz frequency. The output voltages from (**a**) FSR, (**b**) 0 wt.% unpoled, (**c**) 0.2 wt.% unpoled, (**d**) 0.4 wt.% unpoled, (**e**) 0.6 wt.% unpoled films, and magnified plot from a single cycle of press and release (by the shaker) of unpoled 0.6 wt.% CB film shown by the dotted rectangle in (**e**). Clear evidence of damping can be observed from the oscillatory behavior with reducing amplitude.

The generated output voltage response of (PVDF-TrFE)/CB films, varying from 0 to 0.6 wt.%, are shown in Figure 9b–e under periodic force provided by the shaker at 1 Hz frequency, using the setup shown in Figure 8. The force applied by the shaker to the film was determined using an FSR, which utilized a simple voltage divider circuit and was compared with a calibration chart provided by the manufacturer, as shown in Supplementary Figures S5a–c. The maximum output voltages obtained for 0 wt.%, 0.2 wt.%, 0.4 wt.%, and 0.6 wt.% under a 6 N applied force (determined from the FSR response shown in Figure 9a) were found to be 0.5 V, 1 V, 1.9 V, and 3 V, respectively (Figure 9b–e). The enlarged view from the 0.6 wt.% CB composite demonstrates a single cycle of pressing and releasing, accompanied by damping. This is clear evidence of oscillatory behavior in response to the external force applied by the shaker. When subjected to gentle finger tapping, the unpoled 0.6 wt.% composite generates a peak-to-peak output voltage of 3 V (see Supplementary Figure S6). This suggests that this self-poled PENG is suitable for detecting human motions, including touching and walking. We also observed that the poled composites, subjected to 100 V/µm for an hour, exhibited significantly higher output voltages: 3.8 V compared to 0.5 V for the unpoled 0 wt.% CB sample, and 8 V compared to 3 V for the unpoled 0.6 wt.% CB sample. This increase in output voltage is attributed to the highly aligned dipoles resulting from the poling process. Table 3 presents the generated output voltages of the fabricated PENGs with different weight percentages under both poled and unpoled conditions.

**Figure 10:**
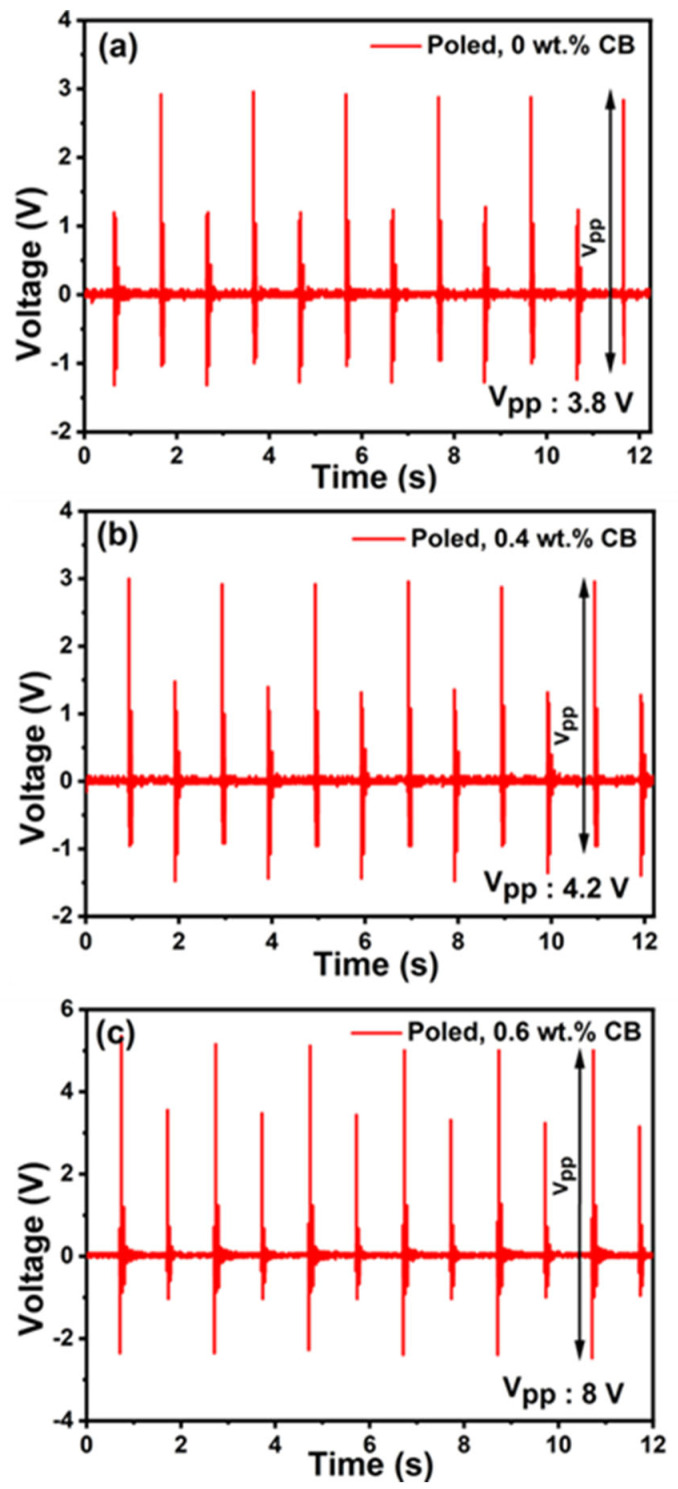
(**a**) Frequency−dependent calibrated output voltage of different poled P(VDF−TrFE)/CB composite films under 6 N applied force and 1 Hz excitation frequency with an active area of 2 × 2 cm. (**a**) The 0 wt.% poled, (**b**) 0.4 wt.% poled, and (**c**) 0.6 wt.% poled.

We found that the unpoled 0.6 wt.% P(VDF-TrFE)/CB composite film produced a maximum peak-to-peak output voltage of 3 V, which is quite comparable to previously reported conductive nanofiller-based PENGs despite our film thickness being much smaller. This resulted in much higher energy densities compared to earlier reports for a similar magnitude of applied force, as shown in Table 4. We utilized unpoled 0.6 wt.% CB film to determine the output power performance with a 1 MΩ load resistance. The piezoelectric power generated by the film can be calculated as P = V^2^/R_L_, where V is the voltage applied to the load resistor R_L_. The unpoled 0.6 wt.% CB composite generated an output voltage and current of 1.5 V and 1.5 µA, respectively, resulting in an output power of 2.25 µW. A detailed comparison of the various performance metrics of the as-fabricated self-poled P(VDF-TrFE)/CB composite film with previously reported results based on other nanocomposite PENGs is included in Table 4 [49,50,51,52,53,54]. We observe that our composite films, with a very low thickness of 5 µm and realized through a facile fabrication process, exhibit the best output voltage per unit thickness and power density, achieving 0.3 V/µm and 1.1 mW/cm^3^ for unpoled films and 1 V/µm and 12 mW/cm^3^ for poled 0.6 wt.% CB composite films, respectively. These figures are several times higher than the best results reported in the literature. The output voltage of poled P(VDF-TrFE)/CB films was measured under the same applied force of 6 N, and the results are shown in Figure 10a–c. As expected, the peak-to-peak output voltage increases for the composite films after poling, reaching 3.8 V and 8 V for 0 and 0.6 wt.% CB films, respectively, as opposed to 0.5 V and 3 V for their unpoled counterparts. However, it is worth noting that the alignment of the dipoles diminishes significantly over time, as our study further indicated.

**Figure 11:**
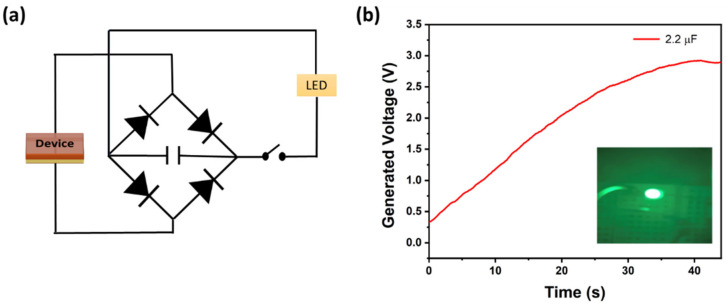
(**a**) Full-wave bridge rectifier circuit for charging the capacitor. (**b**) Charging transient for the 2.2 µF capacitor using an unpoled P(VDF-TrFE)/CB composite film with 0.6 wt.% of CB under applied force (6 N) and 1 Hz frequency. The inset shows a picture of a glowing LED using the harvested energy.

To demonstrate the possible practical application of this self-poled P(VDF-TrFE)/CB-based PENG, a simple full-wave rectifier bridge circuit was designed with an output capacitor (2.2 µF), four diode rectifiers (IN 4007), a switch, and a light-emitting diode (LED, 515–520 nm, and 0.6 µW) as shown in Figure 11a. We also investigated the charging of a 2.2 µF capacitor (C) under the same loading condition, where the output voltage was found to reach a steady state of 2.6 V after 35 s, with 6 N applied force, and the energy stored in the capacitor reached 7.4 µJ (E = ½CV^2^). This stored energy successfully lit up a green LED for a few seconds, as shown in the inset Figure 11b. A video link for this has been added in the Supplementary Section in Figure S7 with an image of glowing RGB color LEDs.

**Figure 12:**
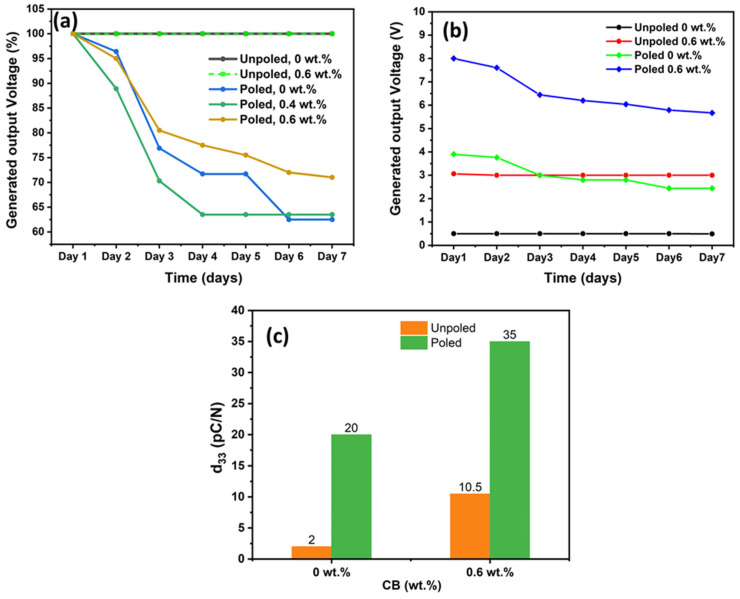
(**a**) Piezoelectrically generated output voltage of poled and unpoled P(VDF-TrFE)/CB composite films measured over a duration of 7 days. (**b**) Comparison of the peak-to-peak output voltage for poled and unpoled CB films over 7 days. (**c**) Bar charts comparing the calculated d_33_ coefficients for 0 and 0.6 wt.% P(VDF-TrFE)/CB composites with and without poling.

To evaluate the loss in piezoelectric performance of the poled composite films over time, we conducted tests for a duration of 7 days. We determined the change in their polarization by measuring the peak-to-peak output voltages as a function of time. Table 5 summarizes the output voltages for the poled and unpoled 0 wt.% and 0.4 wt.% CB samples, as well as the poled 0.6 wt.% CB sample over a one-week period. Figure 12a illustrates the reduction in percentage output voltage for these films to facilitate better comparison. We observed a significant reduction in output voltage (and therefore polarization) for poled composite films over time, resulting in a loss of approximately 27% to 55% of their polarization within 7 days (see Figure 12a). The significant decrease in polarization observed in the poled films aligns with an earlier report [55]. As expected, over the 7-day period, the unpoled film showed no loss in polarization. Figure 12b provides a direct comparison between the 0 and 0.6 wt.% CB composite films under poling and unpoling conditions in terms of the retention of their piezoelectric properties over time.

We observed that the piezoelectric properties of the unpoled 0.6 wt.% CB film remained essentially unchanged even after a week, consistently producing an output voltage of ~3 V. In contrast, the poled 0 wt.% CB film lost 36.8% of its original polarization during the same timeframe, resulting in a reduced output voltage of only 2.4 V compared to the initial 3.8 V immediately after poling. Indeed, as shown in Figure 12b, the output voltage of an unpoled 0.6 wt.% CB film (3 V) remained 0.6 V higher than the unpoled 0 wt.% CB film, which generated 2.4 V after 7 days when the output voltages stabilized. Moreover, poled 0.8 wt.% CB film lost its polarization over time gradually, resulting in a voltage of only 5.2 V. These results clearly indicate that self-aligned dipoles created by the chemical incorporation of additives are much more stable than dipole alignment achieved during poling by applying an electric field.

The d_33_ coefficient is an important indicator of the piezoelectric performance of films and is particularly useful for comparing films of different sizes and thicknesses. It is influenced by various factors, including the degree of poling and the type and content of nanomaterials in composites [16]. In our experimental setup, we measured the applied force to the film using an FSR, as depicted in Figure 8. However, since the exact area of the force application is uncertain, we estimated the variation of the d_33_ coefficient, assuming the average d_33_ values of 0 wt.% CB content film (pure P(VDF-TrFE) film) reported in the literature, and then determining the relative change in d_33_ with CB composition. Hu et al. [44] reported the d_33_ value for annealed P(VDF-TrFE) film without any nanofiller as (3 pC/N), which agrees well with the d_33_ value for the 140 °C annealed film reported by Chen et al. [56] of 2.0 ± 0.8 pC/N. Thus, we assumed a d_33_ value for our 0 wt.% CB film as 2.8 pC/N based on the above reports. The output voltage generated by a piezoelectric film of thickness (l), under a force (F), applied over an effective area A_eff_ is given by the equation

(2) $$V=\frac{d_{33\times l\times F}}{k\times \varepsilon \times Aeff}$$

where d_33_, k, ε are the piezoelectric strain coefficient, dielectric constant of the P(VDF- TrFE), and permittivity, respectively. The effective area (A_eff_) was found to be 1.4 mm^2^, a small fraction of the actual sample area of 4 cm^2^. Most of the parameters on the right-hand side of Equation (2) remain the same for the films, except d_33_ and thickness (which increased slightly with increasing CB content). We can calculate the d_33_ values for composites with various CB percentages. The calculated d_33_ values, both with and without annealing, are plotted in Figure 12c and listed in Table 5. We particularly observed a significant improvement in the d_33_ value, which increased from 2 pC/N to 10.5 pC/N when 0.6 wt.% CB was added to P(VDF-TrFE) under the same fabrication conditions. This structural enhancement is reflected in the much higher intensity of the maximum *β*-phase peak in the XRD spectra, as depicted in Figure 2d. Microscopically, such an enhancement is expected because an increased CB percentage leads to a higher fraction of β-crystallite formation through specific interactions between the hydroxyl groups found in CB and the CF_2_ segments of P(VDF-TrFE). The d_33_ value for P(VDF-TrFE) composite films has been reported by Hu et al. [44] to be 6 pC/N for unpoled P(VDF-TrFE)/GO composites. Similarly, Gwang Ho Kim et al. [57] fabricated PVDF/MWCNT nanocomposites and reported a d_33_ value of 7.5 pC/N. To date, the highest reported d_33_ value has been achieved by Badatya et al. [58] for self-poled PVDF/CNT composite foam, reaching a value of 9 pC/N. However, these d_33_ values are still lower than the d_33_ coefficient of 10.5 pC/N estimated for self-poled 0.6 wt.% CB composite films in this work. As expected, the d_33_ value further increased with poling. For the 0 wt.% CB film, the d_33_ increased sharply from 2 pC/N to 20 pC/N immediately after poling, slightly higher than the d_33_ values reported for poled pure P(VDF-TrFE) in earlier studies, which were 16 and 18 pC/N [16,59]. Similarly, for the 0.6 wt.% CB film, the change is also significant, with d_33_ rising more than three times from 10.5 pC/N to 35 pC/N. Once again, the d_33_ value of our poled 0.6 wt.% CB composite film, measured immediately after poling, surpasses the best-reported d_33_ values of carbon-based nanocomposites to date [42,44,60]. In fact, the d_33_ value is also higher than that of recent inorganic piezoelectric nanoparticle-based P(VDF-TrFE) composites, including Ag, ZnO, and BaTiO3 [61,62,63]. However, the value of d_33_ for both poled 0 and 0.6 wt.% CB films significantly decreased after 7 days, stabilizing at values of 10 pC/N and 18.9 pC/N, respectively, the poled 0.6 wt.% CB film still exhibits a higher d_33_ value compared to the best-reported d_33_ values for poled composite films containing graphene oxide (GO) and carbon nanotubes (CNT) of 10.5, 12.25, and 16 pC/N, respectively [64,65].

Our experimental findings highlight the advantages of piezoelectric composite films with CB, whether poled or not, for practical applications. These advantages include an easy fabrication process, exceptional material properties, thin film design, and high piezo coefficients. This device shows promise for sequentially charging multiple capacitors over time, enabling the storage of substantial energy for applications such as wearable/implantable bio-electronic devices and smart monitoring systems [66,67,68]. They also open up applications in sensing force, strain, and pressure, offering credible competition to existing inorganic piezo-based material sensors, including those based on PZT [69], LiNbO3 [70], and III-Nitrides [71,72,73].

## 5. Conclusions

The composite films were investigated using multiple characterization techniques, demonstrating their high material quality across all CB percentages. The piezoelectric voltage generated via the composite films, under similar applied force, increased monotonically with higher CB concentration for both poled and non-poled films, reaching a peak in piezoelectric voltage generation at 0.6 wt.%, beyond which the films exhibited low resistance due to conducting bridges formed by the CB. At a 0.6 wt.% CB composition, we measured the highest peak-to-peak output voltage of 3 V, which is six times higher than that of the unpoled 0 wt.% CB film. The piezoelectric properties of the unpoled composite films also exhibited excellent stability with time, in contrast to the rapid reduction observed for poled films, leading to superior piezoelectric performance for the unpoled 0.6 wt.% CB film compared to the poled 0 wt.% CB film after a week. The superior piezoelectric performance of the unpoled 0.6 wt.% composite films was further enhanced with poling, resulting in high d_33_ values of 10.5 and 35 pC/N, respectively, which are among the best reported so far for all carbon-based composite P(VDF-TrFE) films.

## Figures and Tables

**Table 1:** Summary of XRD spectra of synthesized 0.6 wt.% CB composite film under different crystallization temperatures (T_cr_).

Temperature (°C)	2theta (θ)	D Spacing (Å)	FWHM	Intensity (cps)
60	19.8	4.46	1.54	2292
70	19.7	4.58	0.81	3366
90	19.4	4.57	0.67	6297
140	19.3	4.59	0.68	9266

**Table 2:** Summary of XRD spectra, FTIR measurements, and AFM-measured surface roughness of different CB wt.% varied from 0 to 1 and annealed at 140 °C.

CB (%)	2θ (°)	D Spacing (Å)	FWHM	Intensity (cps)	F(β) (%)	Roughness (nm)
0	19.34	4.585	0.72	5805	76	5.2
0.2	19.32	4.589	0.71	6589	81	8
0.4	19.33	4.587	0.70	7903	83	21
0.6	19.38	4.591	0.68	9266	96.6	37
0.8	19.35	4.583	0.71	7892	87.4	-
1	19.38	4.590	0.72	4986	74.7	-

**Table 3:** Generated output voltage and d_33_ coefficient value for different poled and unpoled CB wt.% composite films under 1 Hz excitation and 6 N force.

CB wt.%	Poling Condition	V_pk-pk_ (V)	d_33_ (pC/N)
0	Unpoled	0.5	2.8
0.2	Unpoled	1	5.4
0.4	Unpoled	1.9	8.8
0.6	Unpoled	3.0	10.5
0	Poled	3.8	20
0.4	Poled	4.2	21
0.6	Poled	8	35

**Table 4:** Comparison of piezoelectric performance of the PVDF and its copolymer-based composites, including the P(VDF-TrFE)/CB composite film from this work (shown in bold).

Poling	Samples	Synthesis	Size (cm^2^ × μm)	Stimuli	Maximum Voltage (V)	Current (µA)	Power Density (µW/cm^3^)
**Self-poled**	PVDF/MWCNT [19]	Phase inversion	9 × 250	~3.7 N 4.13 kPa	10_pk-pk_	-	-
	PVDF/Yb 3+ [23]	Casting	5 × 170	85 N 0.17 MPa	10	-	5.7
	PVDF/PFD-BTO [24]	Casting	6 × 30	100 N ~166 kPa	5.9	0.3	102
	PVDF-TrFE/rGO [29]	Casting	6 × 21	2 N 3.3 kPa	2.4	1.3	253.3
	PVDF/ZnO [49]	Casting	2.25 × 60	3.5 N 15 kPa	1.8_pk-pk_	0.57	34.8
	PVDF [50]	Quenching	4 × 30	Finger tapping	19.2_pk-pk_	0.7	758
	**This work**	**Spin coating**	**4 × 5.4**	**6 N** **15 kPa**	**1.5**	**1.5**	**1, 100**
**Poled**	PVDF/rGo [44]	Scrap coating	20 × 10	5 N 2.5 kPa	8.3	0.6	28.7
	PVDF-TrFE/MXene [51]	Electrospinning	1 × 40	20 N 0.2 Mpa	1.58_pk-pk_	-	91.1
	PVDF-TrFE/BaTiO_3_ [52]	Printing	1 × 60	50 N 0.5 MPa	13.2	0.3	2200
	PVDF/Graphene [53]	Casting	1.4 × 50	1g	1.3_pk-pk_	-	5.2
	PVDF/BTO [54]	Spray coating	8 × 50	2 N 2.5 kPa	10_pk-pk_	2.5_pk-pk_	145
	**This work**	**Spin coating**	**4 × 5.4**	**6 N** **15 kPa**	**5.1**	**5.1**	**12, 000**

**Table 5:** Generated output voltage (V_pk-pk_) for different P(VDF-TrFE)/CB composite films tabulated with time elapsed after poling with 1 Hz excitation and 6 N force.

Samples	Day1 (V)	Day2 (V)	Day3 (V)	Day4 (V)	Day5 (V)	Day5 (V)	Day7 (V)
Unpoled, 0 wt.%	0.5	0.5	0.5	0.5	0.5	0.5	0.5
Unpoled, 0.6 wt.%	3	3	2.98	3	3	3	3
Poled, 0 wt.%	3.8	3.76	3	2.8	2.83	2.3	2.3
Poled, 0.4 wt.%	4.7	4.2	3.32	3.2	3.1	3.1	3.1
Poled, 0.6 wt.%	8	7.60	6.44	6.2	6.04	5.79	5.2

## Data Availability

The data presented in this study are available on request from the corresponding author.

## Supplementary Materials

The following supporting information can be downloaded at https://www.mdpi.com/article/10.3390/polym15204131/s1, Figure S1: XRD pattern of Pure P(VDF-TrFE) film at (**a**) 60 °C and (**b**)140 °C. At lower crystallization temperature (60 °C), 2ϴ peak position is appeared at 19.7° whereas peak position is shifted to lower value (19.3°) when the P(VDF-TrFE) film sis annealed at 140 °C; Figure S2: FTIR spectra of different CB loaded composite film within the region of 3100 to 2900 cm^−1^. This specific region is contributed to OH bond and symmetric and asymmetric stretching of -CH2 at 3012 cm^−1^ and 2978 cm^−1^, which are not associated with any other vibrational bonds);Figure S3: AFM image (5 × 2.5 µm) of pure P(VDF-TrFE) film annealed at 150 °C. Roughness is prominent when P(VDF-TrFE) film is annealed near melting temperature (Tm). Needle like crystallites were seen and surface roughness of the P(VDF-TrFE) film was dramatically increased to 36.8 nm whereas 140 °C exhibits only 5 nm thickness with smooth surface; Figure S4: Resistivity of various CB loaded composite films over four different lengths along with linear fitting, varying from (**a**) 0.8 wt.% (**b**) 1 wt.% (**c**) 1.5 wt.%, and (**d**) 2 wt.%. Contact resistance and sheet resistance have been calculated using MATLAB. Figure S5: (**a**) Force sensor circuit diagram used to determine applied force from output voltage. (**b**) Calibration chart of FSR provided by the manufacturer. One newton equals to about 98 g. 10 KΩ resistance is used in our circuit. (**c**) Calibrated Force in newton vs. FSR voltage. Video S1: A video shows the demonstration of periodic pressing and releasing on the device and LED glowing; Figure S6: Generated output voltage of unpoled 0.6 wt.% CB composite film under irregular gentle finger tapping. Figure S7: A picture of different color LEDs that glow from drawing the energy from capacitor after unpoled 0.6 wt.% P(VDF-TrFE)/CB composite subjected to an external force (6N) with 1Hz frequency.
