# Supplementary material for: Self-Polarized P(VDF-TrFE)/Carbon Black Composite Piezoelectric Thin Film
Source: Polymers (Basel). 2023 Oct 18;15(20):4131. doi: 10.3390/polym15204131 (PMC10610547; doi:10.3390/polym15204131)
Supplement: Supplementary file 1 [file polymers-15-04131-s001.zip › polymers-2633201-supplementary.pdf]

Supplementary Materials

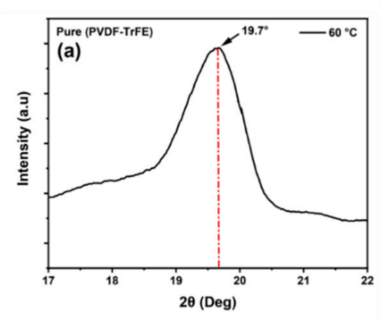

Figure S1. XRD pattern of Pure P(VDF-TrFE) film at (a) 60 °C and (b)140 °C. At lower crystallization temperature (60 °C), 2θ peak position is appeared at 19.7° whereas peak position is shifted to lower value(19.3°) when the P(VDF-TrFE) film sis annealed at 140 °C.

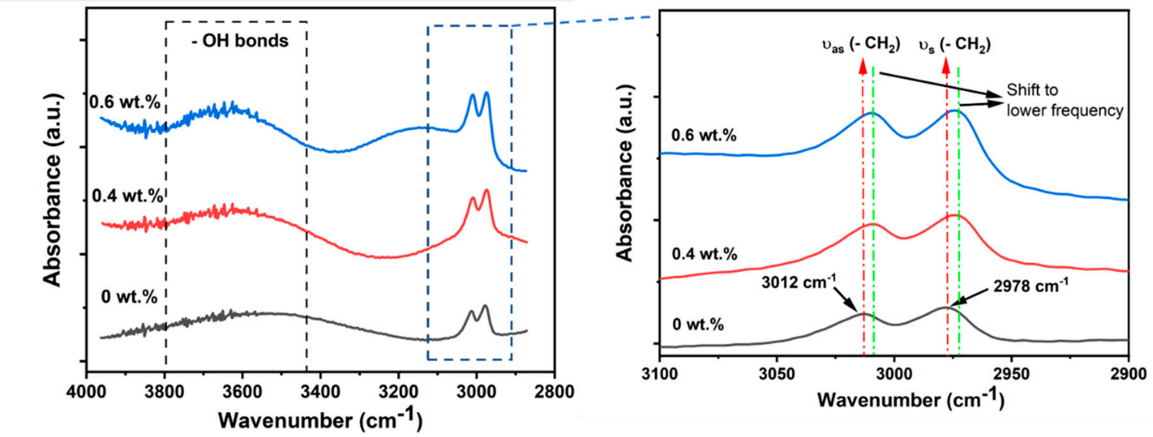

Figure S2. : FTIR spectra of different CB loaded composite film within the region of 3100 to 2900 cm^{-1}. This specific region is contributed to OH bond and symmetric and asymmetric stretching of - CH_2 at 3012 cm^{-1} and 2978 cm^{-1} , which are not associated with any other vibrational bonds).

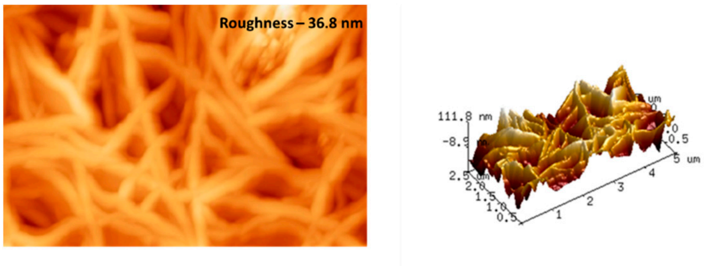

Figure S3. AFM image (5 x 2.5 µm) of pure P(VDF-TrFE) film annealed at 150 °C. Roughness is prominent when P(VDF-TrFE) film is annealed near melting temperature (T_m). Needle like crystallites were seen and surface roughness of the P(VDF-TrFE) film was dramatically increased to 36.8 nm whereas 140 °C exhibits only 5 nm thickness with smooth surface.

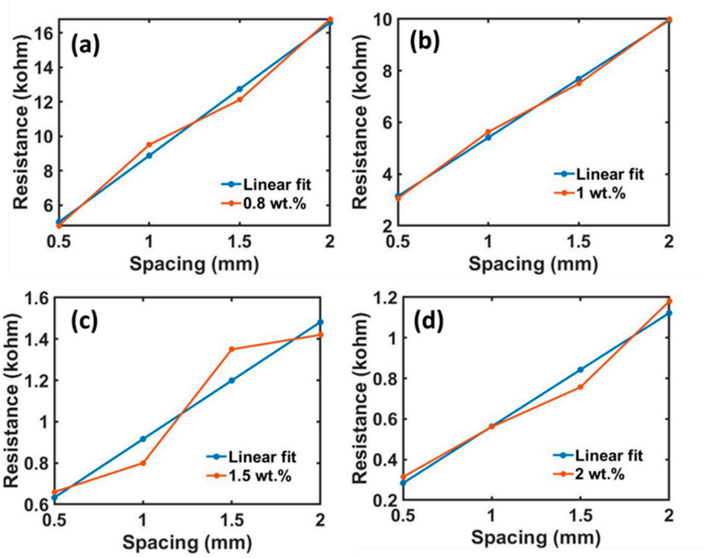

Figure S4. Resistivity of various CB loaded composite films over four different lengths along with linear fitting, varying from (a) 0.8 wt.% (b) 1 wt.% (c) 1.5 wt.%, and (d) 2 wt.%. Contact resistance and sheet resistance have been calculated using MATLAB.

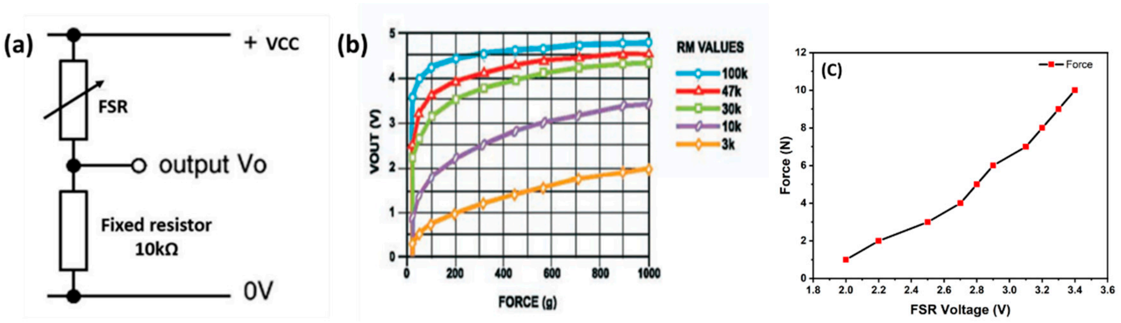

Figure S5. a) Force sensor circuit diagram used to determine applied force from output voltage. b) Calibration chart of FSR provided by the manufacturer. One newton equals to about 98 g. 10 KΩ resistance is used in our circuit. (c) Calibrated Force in newton vs. FSR voltage.

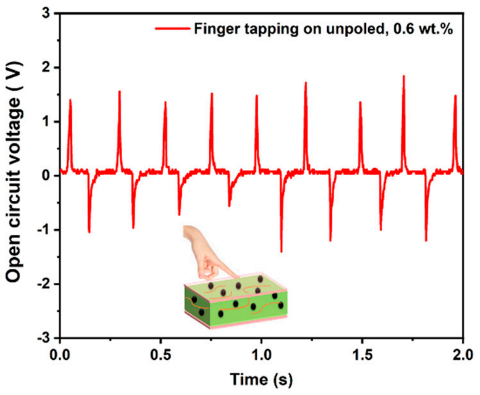

Figure S6. : Generated output voltage of unpoled 0.6 wt.% CB composite film under irregular gentle finger tapping.

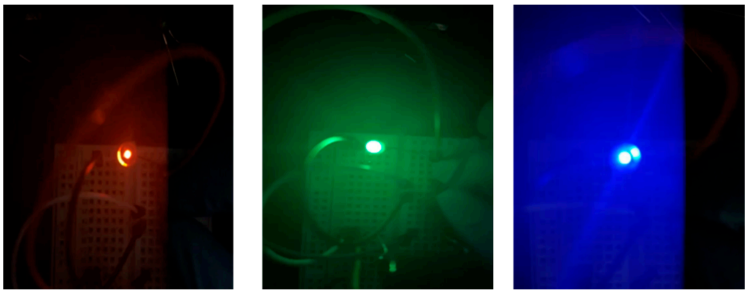

Energy harvesting and LED lightimg up (2).mov

Figure S7. A picture of different color LEDs that glow from drawing the energy from capacitor after unpoled 0.6 wt.% P(VDF-TrFE)/CB composite subjected to an external force (6N) with 1Hz frequency. A video shows the demonstration of periodic pressing and releasing on the device and LED glowing.
